# Impact of lower body mass index on risk of all-cause mortality and infection-related death in Japanese chronic kidney disease patients

**DOI:** 10.1186/s12882-020-01894-7

**Published:** 2020-06-30

**Authors:** Tae Yamamoto, Masaaki Nakayama, Mariko Miyazaki, Hiroshi Sato, Masato Matsushima, Toshinobu Sato, Sadayoshi Ito

**Affiliations:** 1 Division of Nephrology, Endocrinology and Vascular Medicine, Tohoku University Graduate School of Medicine, 1-1 Seiryomachi, Aoba-ku, Sendai, Miyagi 980-8574 Japan; 2 Research Division of Chronic Kidney Disease and Dialysis Treatment, Tohoku University Hospital, 1-1 Seiryomachi, Aoba-ku, Sendai, Miyagi 980-8574 Japan; 3 Department of Internal Medicine, Sendai City Hospital, 1-1 Seiryomachi, Aoba-ku, Sendai, Miyagi 980-8574 Japan; 4 Center for Advanced Integrated Renal Science, Tohoku University Graduate School of Medicine, Sendai, Japan; 5 Department of Nephrology, St. Luke’s International Hospital, Tokyo, Japan; 6 Department of Internal Medicine, JR Sendai Hospital, Sendai, Japan; 7 Department of Clinical Research, The Jikei University School of Medicine, Tokyo, Japan; 8 Kidney Center, Japan Community Health Care Organization Sendai Hospital, Sendai, Japan

**Keywords:** Body mass index, Cause of death, Chronic kidney disease, Infection, Outcome

## Abstract

**Background:**

Several studies have reported that lower body mass index (BMI) is associated with high mortality in patients with chronic kidney disease (CKD). Rate of infection-related death in CKD patients is increasing. However, the relationship between BMI and infection-related death is unclear.

**Methods:**

Overall, 2648 CKD outpatients (estimated glomerular filtration rate < 60 mL/min and/or presenting with proteinuria) under the care of nephrologists were prospectively followed for 5 years. Patients were stratified by quartile of BMI levels. Data on all-cause mortality before progression to end-stage kidney disease (ESKD) and the cause of death were collected.

**Results:**

The median follow-up time was 3.9 years (interquartile range, 1.7–5.0); 114 patients died and 308 started renal replacement therapy. The leading causes of death were as follows; cardiovascular (41%), infection-related (21%), and malignancy-related (18%). Advanced age and lower BMI were the significant risk factors for all-cause mortality before progression to ESKD. Advanced age was statistically associated with respective causes of death, while lower BMI was associated with infection-related death only. CKD stage had no significant impact on all-cause or individual mortality.

**Conclusions:**

Low BMI was associated with significant risk of all-cause mortality and infection-related death, which may indicate the novel clinical target to improve CKD outcomes.

## Background

Chronic kidney disease (CKD) is a risk of all-cause mortality in general population, and the risk is increased by function of progression of CKD stage [1].

Obesity is potentially associated with a higher incidence of cardiovascular events and kidney disease progression in the population-based study [2, 3]. However, interestingly, in patients with CKD, overweight individuals have a lower risk for cardiovascular as well as non-cardiovascular death [4]. And it is revealed that there is an adverse association between body mass index (BMI) and mortality in those with CKD [2, 5], indicating that low BMI is at risk of poor prognosis in CKD. On the other hand, accumulative surveys are indicating the infectious disease as an emerging critical issue in terms of non-cardiovascular mortality for CKD patients [6]; it has been reported that there is a high incidence of hospitalization by infectious disease [7], an increased risk of death by non-cardiovascular mortality including infection-related death [8–10], and the infection as the recent leading cause of death among incident dialysis patients in Japan [11].

Upon the clinical background, we think it is very crucial to examine the association among CKD outcomes, BMI, and the cause of mortality including infection-related death, in order to present the risk stratification of respective patients. The aim of our study was to determine the primary causes of death in Japanese pre-dialysis CKD outpatients, and to examine those possible associations with BMI levels.

## Methods

### Participants — the Gonryo CKD cohort

The Gonryo study is a prospective observational cohort study of patients with CKD treated by nephrologists in high-standard facilities. Our Gonryo CKD project involved outpatients treated in 11 hospitals offering specialized nephrology services, covering almost the entire medical network area of Miyagi, a prefecture located in the northeast area of Japan [12, 13]. The study originally enrolled every outpatient treated between May 2006 and November 2008. A total of 4015 patients provided informed consent for participation. The study protocol was approved by the Institutional Review Boards of Tohoku University School of Medicine (approval number 2006–10, UMIN000011211), and of the respective participating hospitals.

**Fig. 1:**
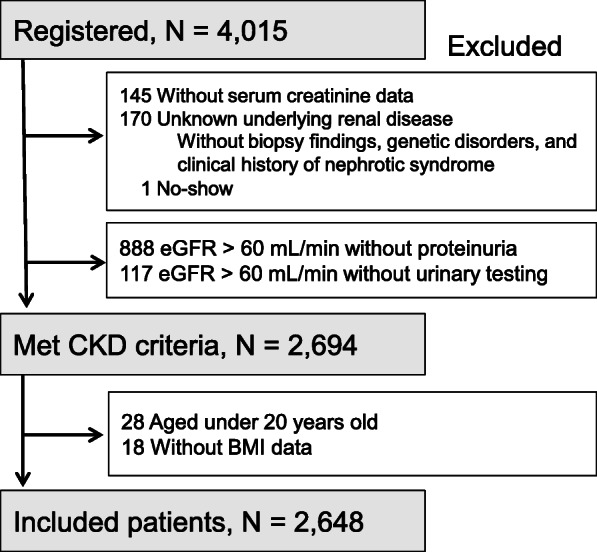
Flowchart of patient inclusion into the present study. The study included outpatients with chronic kidney disease (CKD) under the care of a nephrologist, with defined estimated glomerular filtration rate (eGFR) and proteinuria

Based on previous studies [12, 13], certain participants were excluded from the present analysis for the following reasons (Fig. 1): lack of data on serum creatinine levels (*n* = 145); unknown underlying renal disease (*n* = 170); loss during follow-up (*n* = 1); estimated glomerular filtration rate (eGFR) > 60 mL/min/1.73 m^2^, without proteinuria (*n* = 888); incomplete results of the urine test (*n* = 117); age < 20 years (*n* = 28); or lack of BMI data (*n* = 18). The participants were stratified by quartile of BMI to Q1-Q4. Additional stratifications were based on the age of each group, non-elderly (20–64 years), early-elderly patients (65–74 years), and late-elderly patients (≥75 years), on albumin levels at 4 g/dL, and on CKD stage defined in terms of eGFR levels: ≥60 mL/min/1.73 m^2^ (G1–G2); 59–45 mL/min/1.73 m^2^ (G3a); 44–30 mL/min/1.73 m^2^ (G3b); 29–15 mL/min/1.73 m^2^ (G4); and < 15 mL/min/1.73 m^2^ (G5). Mean blood pressure (MBP), calculated by (systolic blood pressure + 2 × diastolic blood pressure)/3, was stratified into quartile groups. We defined a patient as having diabetes not only if the patient was diagnosed with diabetic nephropathy as the underlying renal disease, but all patients treated for diabetes.

### Outcome measures

The Gonryo study originally aimed to clarify the crucial clinical events among pre-dialysis patients [12]. The primary outcomes were all-cause mortality and progression to end-stage kidney disease (ESKD), as defined by initiation of renal replacement therapy (RRT), which indicated the end of the study follow-up period. Based on the definition of primary events, progression to ESKD is a censer event for all-cause mortality. Study outcomes were collected from the records of annual check-ups, conducted through 5 years after enrollment in the study. The outcomes were determined based on medical records, death certificates, and interviews with the attending physicians at the time of the annual check-ups. Cause of death was defined according to the 10th revision of the International Classification of Diseases (ICD-10) for primary cause of death and categorized as cardiovascular disease (CVD) (disease of the circulatory system: ICD-10 codes I00-I99), infection (A00-B99, J00-J22), malignancy (C00-D48), renal failure (N18) or all other-causes. CVD-related death included death due to ischemic heart disease, congestive heart failure, stroke, or vascular disease. The diagnosis of stroke was based on computed tomography or brain magnetic resonance imaging and was established according to the Classification of Cerebrovascular Diseases III, published by the National Institute of Neurological Disorders and Stroke [14]. Infection-related death included pneumonia, influenza, sepsis, and organ infections considered to have been the case in the category “other”.

### Data collection

Baseline data included patient characteristics and laboratory data extracted from the medical records maintained by each hospital, as previous described [12, 13]. Serum creatinine levels were measured annually using an enzyme assay method at respective hospitals, and the eGFR levels (mL/min/1.73 m^2^) were calculated based on the formula developed for the Japanese population [15]. Proteinuria was defined as a positive dipstick test in spot urine, which corresponded to a protein concentration of > 30 mg/dl, as measured using the urine analyzer in each hospital. Blood pressure data was obtained using an automatic sphygmomanometer and applying the Korotkoff sound technique with the patient in a sitting position, and it was not requested to measure several times in the study design. Information regarding ongoing treatments was obtained from the medical records.

### Statistical analysis

Data were described in terms of absolute range, percentage, or median with an interquartile range. A *P*-value < 0.05 indicated statistical significance. Differences between groups were analyzed using a t-test or one-way analysis of variance (ANOVA); if a relationship demonstrated significance on the ANOVA test, a post-hoc test was applied. Correlations were calculated using the non-parametric Spearman rank test. In the survival analysis, the incidence rate was calculated as the number of events per person-time, survival probability was estimated using the Kaplan-Meier method, and the results were compared using the log-rank test. The risk for all-cause mortality before progression to ESKD was calculated using the competing-risk approach, as the patients who began RRT were not followed up as part of this study; the results are presented as hazard ratios with 95% confidence intervals. The multivariate analyses were adjusted for age, sex, BMI, smoking, albumin, diabetes, CVD history, MBP, CKD stage, and proteinuria. The test of interaction was estimated by adding multiplicative interaction terms between BMI levels and CKD stages, age, or sex, however the variables not affected significantly on the association between BMI and the risk of all-cause mortality. Since the incidence rate of each cause of death was limited, we adjusted for age, BMI and two other selected variants in multivariate models. All data were analyzed using Stata version 15.1 (Stata Corp LP, College Station, Texas, USA).

## Results

### Baseline characteristics by BMI group

Among the total 2648 CKD outpatients with CKD evaluated in the present study, the median /mean with standard deviation of BMI was 23.6/23.8 ± 3.5 kg/m^2^ in males and 22.6/23.1 ± 4.1 kg/m^2^ in females. In the overall study population of patients with CKD, the median age was 63 years (range, 51–73 years), 53.5% of the patients were male, and the median eGFR at baseline was 53.4 mL/min/1.73 m^2^ (range, 33.1–73.2 mL/min/1.73 m^2^). The characteristics of the participants, stratified according to BMI group, are summarized in Table 1.

**Table 1:** Characteristics of patients with chronic kidney disease, stratified according to BMI group

BMI group	Q1	Q2	Q3	Q4	*P*
N	661	662	663	662	
BMI (kg/m^2^)	19.5, 18.4–20.3	22.0, 21.5–22.6	24.3, 23.7–24.8	27.6, 26.5–29.6	
Age (y)	59, 42–73	65, 54–74	63, 55–72	61, 51–71	<.0001
Gender (male, %)	40.8	55.0	61.2	56.9	<.0001
Body high (cm)	159, 153–165	161, 154–167	162, 154–168	161, 153–168	0.0012
Body weight (kg)	48.5, 44–53.5	57, 52–62	63, 57.5–69	73, 65–80	<.0001
Smoking (%)	16.4	15.6	16.6	16.5	0.961
Albumin (g/dL)	4.0, 3.7–4.3	4.0, 3.7–4.3	4.1, 3.8–4.3	4.1, 3.9–4.4	<.0001
Hemoglobin (g/dL)	12.3, 10.8–13.4	12.7, 11.2–13.9	13.3, 11.6–14.6	13.6, 12.1–14.9	<.0001
Diabetes (%)	18.8	27.6	32.9	33.8	<.0001
History of CVD (%)^a^	16.3	17.1	20.2	15.3	0.097
Stroke (%)	6.5	6.7	8.1	4.7	0.087
Cardiac disease (%)	11.8	12.1	14.3	11.5	0.383
SBP (mmHg)	125, 116–137	130, 121–140	132, 122–141	132, 124–142	<.0001
DBP (mmHg)	74, 66–81	77, 70–82	77, 70–84	80, 70–86	<.0001
MBP (mmHg)	92, 84–99	94, 88–101	96, 88–102	97, 90–104	<.0001
CKD stage					0.276
G1–2	43.4	37.6	38.3	40.0	
G3a	18.5	22.7	22.9	24.2	
G3b	14.4	16.5	16.9	15.1	
G4	14.7	14.5	12.2	13.1	
G5	9.1	8.8	9.7	7.6	
eGFR (mL/min/1.73 m^2^)	54.7, 31.6–77.1	52.0, 31.7–71.6	52.8, 33.6–69.8	55.1, 35.8–72.6	0.149
Proteinuria (%)	44.9	49.0	50.2	55.2	0.003

Data were described in terms of absolute range, percentage, or median with interquartile range. A *P*-value < 0.05 indicated statistical significance. Abbreviations: *BMI* body mass index, *N* number of patients, *CVD* cardiovascular disease, *SBP* systolic blood pressure, *DBP* diastolic blood pressure, *MBP* mean blood pressure, *CKD* chronic kidney disease, *eGFR* estimated glomerular filtration rate

### Incidence of primary outcomes by BMI group

**Fig. 2:**
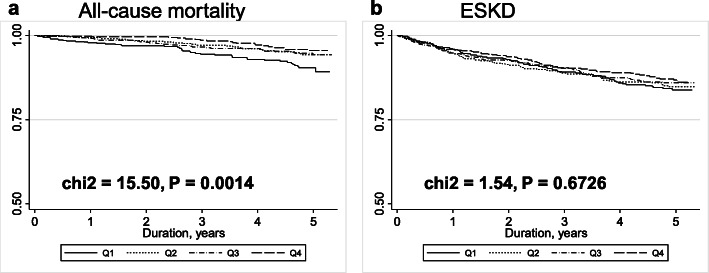
Survival curves for patients with chronic kidney disease according to BMI group. Among 2648 patients with CKD, 114 patients died and 308 patients progressed to ESKD. Kaplan-Meier survival curves showed that the survival risk was significantly associated with the lower BMI group (log-rank χ^2^ = 15.5, *P* = 0.0014), but the incidence of ESKD did not differ between the defined BMI groups

During a median follow-up of 4.63 years (range, 1.88–5.00 years), 114 patients died, and 308 patients progressed to ESKD. While the incidence of ESKD did not differ between the defined BMI groups (log-rank χ^2^ = 1.54, *P* = 0.673), the survival risk before progression to ESKD was significantly associated with the lower BMI group (log-rank χ^2^ = 15.5, *P* = 0.0014) (Fig. 2). This association was evident in patients with physical wasting, such as in those over 75 years-old (Log-Rank χ^2^ = 210.57, *P* < 0.0001) or in those with albumin levels less than 4.0 mg/dl (Log-Rank χ^2^ = 58.70, *P* < 0.0001), respectively (Supplemental Figure 1).

### Frequency for each category of mortality

**Fig. 3:**
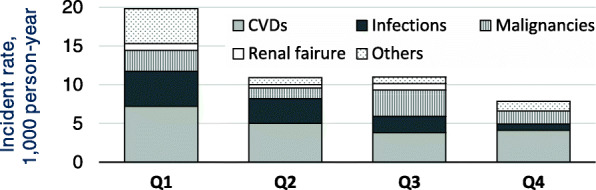
The incidence rate of each cause of death, according to BMI group. The event rates, per 1000 persons per year, for cardiovascular, infection-related, and malignancy-related incidence are presented. The incidence rates for infectious-related death show a clear association with BMI classification

Among the 114 deaths, the details of each which are summarized in Table 2, the most common cause was CVD (41%), followed by infection (21%) and malignancy (19%). While CVD and malignancy were observed in all generations, infection-related death was observed only in the elderly (over 65 years-old), as 25% in early-elderly patients and 75% late-elderly patients. When each cause varies by BMI group, the incident of death, including CVD and infection-related, increased in the lowest BMI group (Fig. 3).

**Table 2:** Details on each mortality cause among 114 deaths, and the incidence rate (per 1000 person-years) among 2648 patients with chronic kidney disease

		All patients			BMI group, (N)			
**Cause of death**	**ICD-10 classification**	**(N)**	**Incidence Rate**	**95% CI.**	**Q1**	**Q2**	**Q3**	**Q4**
**Cardiovascular**	Stroke	11	358.7	198.6–647.6	2	5	3	1
	Cardiac disease	29	374.1	260.0–538.3	12	5	6	6
	Diseases of arteries, arterioles and capillaries	7	366.7	164.8–816.3	2	1	1	3
**Infectious-related**	Infections	24	446.6	299.3–666.3	10	7	5	2
**Malignancy-related**	Malignancies	21	367.0	239.3–562.9	6	3	8	4
**Uremia**	Chronic kidney disease	5	322.2	134.1–774.1	2	1	2	0
**Others**	Other	17	386.0	240.0–621.0	10	2	2	3
**Total**	–	114	383.0	318.5–460.5	44	24	27	19

The incidence rate and number of events (N) for the primary outcomes and for each cause of death is given in *1000 person-years.* The incidence rate for cardiovascular disease included stroke, cardiac disease, and vascular disease. Uremia is defined as: main cause of death is end-stage renal disease withholding renal replacement therapy. The incidence rate and number of events (N) according to BMI group is also presented. Abbreviations: *ICD-10* the 10th revision of the International Classification of Diseases, *95% CI* 95% confidence interval, *BMI* body mass index

### Risk factors for all-cause mortality and progression to ESKD in elderly patients with CKD

Crude and multivariate models of the risk for primary outcomes are presented in Table 3. The progression to ESKD was not associated with BMI. However, the risk for all-cause mortality before progression to ESKD in the lower BMI group was 1.26 times higher than in the higher BMI group. In the multivariate competing-risk analysis, the risk of all-cause mortality significantly increased with age, lower BMI, smoking, history of CVD, and lower MBP, but was not associated with renal function or proteinuria.

**Table 3:** Crude and adjusted competing-risk analysis for all-cause mortality and end-stage kidney disease (ESKD) in patients with chronic kidney disease

	*All-cause mortality*				*ESKD*			
Univariate	SHR	95% CI		*P*	SHR	95% CI		*P*
BMI group, lower	**1.34**	**1.13**	**1.60**	**0.001**	**1.05**	**0.96**	**1.16**	**0.293**
Multivariate	SHR	95% CI		*P*	SHR	95% CI		*P*
Age group, elder	**2.88**	**2.17**	**3.83**	**<.001**	0.88	0.74	1.04	0.120
Gender, male	1.30	0.86	1.96	0.208	**1.46**	**1.10**	**1.93**	**0.009**
BMI group, lower	**1.26**	**1.04**	**1.52**	**0.017**	1.03	0.92	1.15	0.658
Albumin, < 4 g/dl	1.40	0.91	2.16	0.124	**2.06**	**1.57**	**2.68**	**<.0001**
Smoking, yes	**1.87**	**1.14**	**3.08**	**0.014**	**1.40**	**1.00**	**1.94**	**0.047**
Diabetes, yes	1.19	0.80	1.77	0.393	**1.52**	**1.16**	**2.01**	**0.003**
CVD history, yes	**2.75**	**1.85**	**4.10**	**<.001**	**0.60**	**0.44**	**0.83**	**0.002**
MBP group, higher	**0.81**	**0.66**	**0.98**	**0.030**	**1.16**	**1.03**	**1.31**	**0.013**
CKD stage, higher	1.06	0.91	1.23	0.483	**6.34**	**5.21**	**7.71**	**<.0001**
Proteinuria, yes	1.27	0.81	2.00	0.301	**2.83**	**1.87**	**4.27**	**<.0001**

Crude and adjusted competing risks for all-cause mortality and ESKD were calculated. BMI Q4 was used as reference. Bold font indicates statistical significance (*P* < 0.05). Covariates in multivariable-adjusted models included age, sex, serum albumin, smoking, diabetes, history of cardiovascular disease, mean blood pressure, CKD stage stratifications, and proteinuria. Abbreviations: *SHR* subdistribution hazard ratio, *95% CI* 95% confidence interval, *BMI* body mass index, *CVD* cardiovascular disease, *MBP* mean blood pressure, *CKD* chronic kidney disease

The risks of each common cause of death were also examined. As expected, advanced age was the primary risk factor for all three causes of death. The lower BMI levels were also significantly associated with infection-related death in model 1 (HR: 1.49 [95% confidence interval (CI), 1.00–2.20] and different models. However, CVD and malignancy-related deaths were not associated with BMI (Table 4). In addition to BMI, albumin < 4.0 g/dl was also a critical risk factor in the multivariate model (HR: 2.78 [95% CI, 1.11–6.94]).

**Table 4:** Cox proportional hazard ratios for mortality due to each cause of death, and the association with body mass index and history of cardiovascular disease in patients with chronic kidney disease

	*CVDs*				*Infections*				*Malignancies*			
Univariate	SHR	95% CI		*P*	SHR	95% CI		*P*	SHR	95% CI		*P*
BMI group, lower	1.23	0.94	1.62	0.130	**1.62**	**1.12**	**2.35**	**0.010**	1.05	0.73	1.53	0.782
Model 1	SHR	95% CI		*P*	SHR	95% CI		*P*	SHR	95% CI		*P*
Age group, elder	**3.17**	**2.01**	**4.99**	**<.0001**	**6.37**	**3.43**	**11.81**	**<.0001**	**3.22**	**1.74**	**5.96**	**<.0001**
BMI group, lower	1.14	0.86	1.52	0.368	**1.49**	**1.00**	**2.20**	**0.048**	0.99	0.67	1.46	0.952
CVD history, yes	**3.86**	**2.03**	**7.33**	**<.0001**	**2.82**	**1.24**	**6.42**	**0.013**	1.91	0.68	5.40	0.222
Smoking, yes	**2.82**	**1.32**	**6.05**	**0.008**	2.22	0.73	6.81	0.161	2.79	1.00	7.84	0.051
Model 2	SHR	95% CI		*P*	SHR	95% CI		*P*	SHR	95% CI		*P*
Age group, elder	**2.80**	**1.82**	**4.31**	**<.0001**	**5.82**	**3.10**	**10.93**	**<.0001**	**2.90**	**1.49**	**5.65**	**0.002**
BMI group, lower	1.16	0.88	1.53	0.290	**1.46**	**1.02**	**2.10**	**0.039**	1.00	0.69	1.44	0.983
CVD history, yes	**3.55**	**1.90**	**6.64**	**<.0001**	**2.73**	**1.22**	**6.11**	**0.015**	1.83	0.67	4.99	0.240
MBP group, higher	0.79	0.60	1.03	0.081	0.65	0.39	1.07	0.090	0.78	0.52	1.18	0.245
Model 3	SHR	95% CI		*P*	SHR	95% CI		*P*	SHR	95% CI		*P*
Age group, elder	**3.44**	**2.34**	**5.08**	**<.0001**	**7.05**	**3.88**	**12.83**	**<.0001**	**3.52**	**1.80**	**6.91**	**<.0001**
BMI group, lower	1.21	0.91	1.60	0.192	**1.56**	**1.05**	**2.33**	**0.029**	1.12	0.75	1.67	0.576
CKD stage, higher	1.07	0.88	1.32	0.493	1.03	0.78	1.36	0.848	0.89	0.68	1.17	0.392
Proteinuria, yes	**2.05**	**1.01**	**4.16**	**0.046**	1.80	0.72	4.49	0.209	1.03	0.38	2.85	0.947

Adjusted risks for all-cause mortality and the cause specific mortality were calculated. BMI Q4 was used as reference. Bold font indicates statistical significance (*P* < 0.05)

Covariates in multivariable-adjusted models included age, body mass index, and additional two factors based on different models. Abbreviations: *SHR* subdistribution hazard ratio, *95% CI* 95% confidence interval, *BMI* body mass index, *CVD* cardiovascular disease, *MBP* mean blood pressure, *CKD* chronic kidney disease

## Discussion

The present study clarified the cause of death in Japanese pre-dialysis CKD outpatients under the care of nephrologists, with a specific focus on the impact of BMI on the cause mortality. Our data suggests that a lower BMI is the critical risk for all-cause mortality, and infection-related death.

Prior to our work, numerous studies have shown that an “obesity paradox” exists, where higher BMI is paradoxically associated with better survival in chronic dialysis patients, which is inconsistent with many findings in the general population [2, 5, 16]. Likewise, no significant association is found between higher BMI levels and mortality in patients with advanced CKD with eGFR< 30 ml/min per 1.73 m^2^ (CKD stages 4 and 5) [17]. And as regards to the cause of mortality of CKD, no association was found between high BMI and CVD-related mortality [5]. According to the meta-analysis [2, 5], the risk of death in CKD stages 3–5, is reduced 1% for every 1 kg/m^2^ increase in BMI. On the other hand, the significant impact of low BMI on the patients’ prognosis has been reported in CKD [2, 5], including the present study. Recently, Navaneethan et al. reported that being underweight results in a greater risk for CVD-related, as well as non-CVD -related deaths in CKD [4]. In the present study, it was confirmed that lower BMI was a significant risk for all-cause mortality, and for infection-related death, as well. These findings may indicate the clinical relevance of non-CVD mortality, especially infectious disease, in patients with CKD with lower BMI.

As for the medical background of lower BMI and infection-related death in CKD, it is possible to speculate that age-related wasting may be, at least partly, involved with the increase of infection-related mortality. In the present study, the impact of lower BMI on mortality was most prevalent in the late-elderly group (age more than 75 years old), and in those with serum albumin less than 4 g/dl by Kaplan-Meier analysis (supplemental data). Since we do not have data on the nutritional status except for serum albumin, we cannot present the association of wasting for the events in this study. This issue of muscle wasting needs to be clarified. Nevertheless, in this study, lower BMI was revealed to be independent risk factor for all-cause mortality and infection-related death, even after adjusting for confounding factors including elderly age, and serum albumin level. Therefore, in terms of biological aspect, low BMI may be primary connected with the susceptible background to infectious disease. Recent studies have revealed the skeletal muscle plays as a major immune regulatory organ by generating myokines which have anti-inflammatory and immunoprotective effects [18, 19]. This pathological involvement in subjects with lower BMI needs to be calrified in future in terms of muscle volume of low BMI in CKD.

Regarding the cause of mortality in CKD patients, CVD and/or malignancy-related death have been reported as the leading cause of death in some countries [4, 8, 10]. Interestingly, the cause of death of CKD patients in the present study (the leading cause, cardiac 41% of all death, followed by infections 21.0%, malignancies 18.4%, and stroke 9.6%), was different from the general Japanese population, i.e. malignancy is the leading cause of death [20]. Rather, the profile of death in the present CKD patients was similar to those of undergoing chronic dialysis treatment of Japan (heart failure 24% of all death, followed by infection 21%, malignancy 9%, and stroke 6%, as for 2017, respectively) [11]. Taken together these data, it is speculated that the cause of mortality of CKD may be different by race, nation, and age of the cohort, e.g. racial difference in incidence of cardiac events (less events in Japanese as compared to Caucasian population), rapidly aging society (growing number of aging patients with ESKD in Japan). Therefore, we think that it is crucially important to take into account these background in order to compare the CKD epidemiology.

### Limitations

There are several limitations in the current study. First, we did not measure waist circumstance, body compositions, nutritional status, or frailty score. Using BMI alone is problematic for assessing malnutrition or wasting, since BMI in advanced patients with CKD can be substantially influenced by overall water volume. Second, in our assessment of renal function, we did not consider cystatin levels based on the creatinine levels which can be affected by malnutrition. Third, considering the incident number was limited, we included a few variants in multivariate models for each cause of death. In addition, our observational study design did not provide adequate statistical power to determine the ideal BMI levels that would offer protection against negative outcomes. Furthermore, this study enrolled outpatients receiving nephrology care, which may not represent the general elderly population with CKD.

## Conclusions

Low BMI was associated with significant risk of all-cause mortality and infection-related death in this study. Although there are several limitations in the current study, the observation may highlight the infection-related death as emerging critical issue, and indicate the novel clinical target to improve CKD outcomes.

## Supplementary information

**Additional file 1 **: **Figure S1.** Survival curves for all-cause mortality stratified by age and serum albumin groups. Kaplan-Meier survival curves for all-cause mortality were plotted in 2648 patients with CKD, stratified (A) by age groups: non-elderly (20–64 years), early-elderly patients (65–74 years) and late-elderly patients (≥75 years), and (B) by those with a serum albumin level at 4 mg/dL (χ^2^ = 1.76, *P* = 0.415). The higher rates of all-cause mortality were clearly observed in the patients who experienced wasting (red), such as in the late-elderly patients (log-rank χ^2^ = 50.06, *P* < 0.001) and in those with a serum albumin level lower than 4 mg/dL (log-rank χ^2^ = 50.06, *P* < 0.001), compared to the other groups (black).

## Data Availability

All data were presented in the manuscript.

## Abbreviations

BMI: Body mass index
CI: Confidence interval
CKD: Chronic kidney disease
CVD: Cardiovascular disease
DBP: Diastolic blood pressure
eGFR: Estimated glomerular filtration rate
ESKD: End-stage kidney disease
ICD-10: The 10th revision of the International Classification of Diseases
MBP: Mean blood pressure
N: Number of patients
RRT: Renal replacement therapy
SBP: Systolic blood pressure
SHR: Sub distribution hazard ratio
