# Supplementary figures and images for: Impact of lower body mass index on risk of all-cause mortality and infection-related death in Japanese chronic kidney disease patients
Source: BMC Nephrol. 2020 Jun 30;21:244. doi: 10.1186/s12882-020-01894-7 (PMC7325015; doi:10.1186/s12882-020-01894-7)

## Slide 1
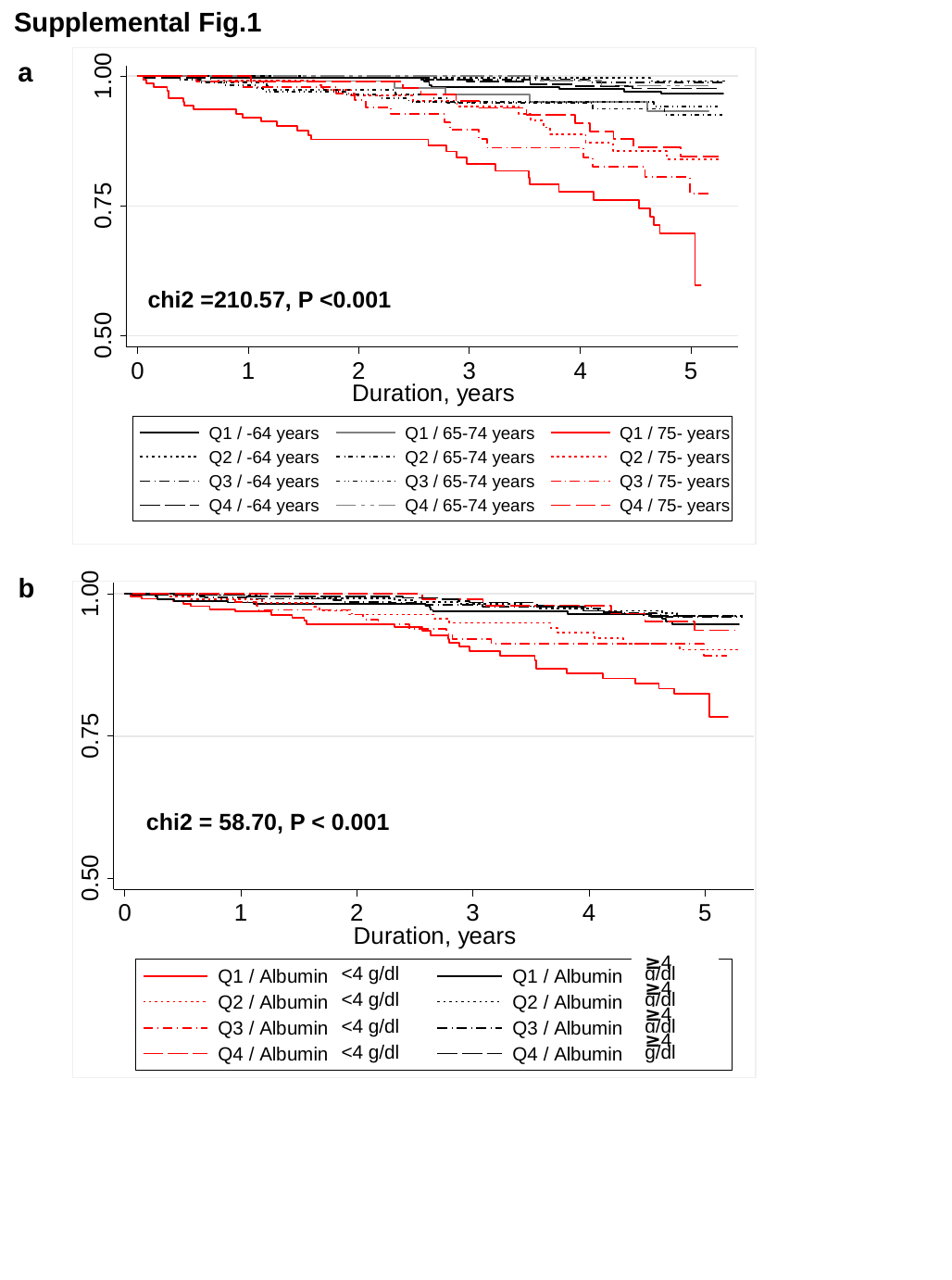

# Supplemental Fig.1
a
 chi2 =210.57, P <0.001
b
 chi2 = 58.70, P < 0.001
<4 g/dl
≥4 g/dl
<4 g/dl
≥4 g/dl
<4 g/dl
≥4 g/dl
<4 g/dl
≥4 g/dl

Supplement: Supplementary file 1 — Additional file 1 : Figure S1. Survival curves for all-cause mortality stratified by age and serum albumin groups. Kaplan-Meier survival curves for all-cause mortality were plotted in 2648 patients with CKD, stratified (A) by age groups: non-elderly (20–64 years), early-elderly patients (65–74 years) and late-elderly patients (≥75 years), and (B) by those with a serum albumin level at 4 mg/dL (χ2 = 1.76, P = 0.415). The higher rates of all-cause mortality were clearly observed in the patients who experienced wasting (red), such as in the late-elderly patients (log-rank χ2 = 50.06, P < 0.001) and in those with a serum albumin level lower than 4 mg/dL (log-rank χ2 = 50.06, P < 0.001), compared to the other groups (black). [file 12882_2020_1894_MOESM1_ESM.pptx]
